# From Genetics to Genomics of Epilepsy

**DOI:** 10.1155/2012/876234

**Published:** 2012-05-08

**Authors:** Silvio Garofalo, Marisa Cornacchione, Alfonso Di Costanzo

**Affiliations:** Dipartimento di Medicina e Scienze per la Salute (Me.S.pe.S.), Università del Molise, Via De Sanctis snc, 86100 Campobasso, Italy

## Abstract

The introduction of DNA microarrays and DNA sequencing technologies in medical genetics and diagnostics has been a challenge that has significantly transformed medical practice and patient management. Because of the great advancements in molecular genetics and the development of simple laboratory technology to identify the mutations in the causative genes, also the diagnostic approach to epilepsy has significantly changed. However, the clinical use of molecular cytogenetics and high-throughput DNA sequencing technologies, which are able to test an entire genome for genetic variants that are associated with the disease, is preparing a further revolution in the near future. Molecular Karyotype and Next-Generation Sequencing have the potential to identify causative genes or loci also in sporadic or non-familial epilepsy cases and may well represent the transition from a genetic to a genomic approach to epilepsy.

## 1. Introduction

In the last decades a large number of gene discoveries have changed our views of idiopathic and symptomatic epilepsy [1]. Indeed, idiopathic epilepsy has the considerable genetic advantage to be found very often in informative autosomal dominant families that have been of great relevance to map and to positional clone the causative gene, opening insight into the biology and molecular pathology of this condition [2, 3].

The search of epilepsy genes has allowed the identification of several genes in idiopathic generalized epilepsy (Table 1), the vast majority of which are channelopathies [4, 5] or affect the activity of excitatory or inhibitory neurotransmitters in central nervous system [6]. It is possible that the dominant nature of these genes due to the multisubunit composition of the molecules have greatly overestimated the role of their mutations in the disease.

Other important insights came from the discoveries of causative genes of syndromic epilepsy (Table 2) [7] and other disorders where epilepsy is associated with encephalopathies (Table 3) [8], mental retardation with brain malformation (Table 4) [9, 10], other neurologic conditions including neuronal migration disorders (Table 5) [11], and inborn errors of metabolism (Tables 6 and 7) [12, 13]. Without any doubt, these discoveries have been great advances in the field; however, their impact on the management of epileptic patients was limited because of the failure to collect significant genetic information from each patient to distinguish the large number of genetic defects that can lead to the disease. Therefore, genetic testing was possible only for few or selected family cases. 

Technical improvements in human chromosomes recognition and better definition of chromosome regions realized by increasing the number of detectable chromosome bands have provided higher resolution of normal and pathological karyotype. It is today well established an association between epileptic seizures and chromosome abnormalities recognized by high-resolution chromosome banding [14, 15]. However, the type and the size of the chromosome defects are not always easy to detect even by the highest-resolution cytogenetic techniques available for light microscopes.

The identification of the specific genetic defect in a patient with epilepsy may clarify the diagnosis (diagnostic testing), suggest the prognosis, assist with treatment and management (e.g., the use of a ketogenic diet in glucose transporter type 1 deficiency syndrome or the avoidance of lamotrigine, phenytoin, and carbamazepine in Dravet syndrome), elucidate the risk of a disease in family members and future children, and save the patient from further diagnostic evaluation and potentially invasive testing.

In asymptomatic subjects with increased risk of seizures because of a family history, genetic test may predict onset of epilepsy (predictive testing) [16, 17]. Despite such potential benefits, genetic testing has also potential harms, such as its ethical, legal, and social implications, and the potential for stigma, distress, adverse labeling, and nonconfidentiality that exists in the setting of inadequate safeguards against discrimination [18]. Considering that our understanding of the epidemiology and clinical utility of genetic testing in the epilepsies is incomplete, the assessment of these potential benefits and harms is particularly complex and is closely linked to the clinical scenario.

The International League Against Epilepsy (ILAE) Genetic Commission presented a tool in the approach to specific tests for epilepsy [16]. According to ILAE report, the diagnostic genetic testing is “very useful” in individual affected by early-onset spasms, X-linked infantile spasms, Dravet and related syndromes, Ohtahara syndrome, epilepsy and mental retardation limited to females, early-onset absence epilepsy, autosomal dominant nocturnal frontal lobe epilepsy, and epilepsy with paroxysmal exercise-induced dyskinesia; the predictive testing is “very useful” in unaffected relatives of individuals affected by Dravet syndrome and epilepsy and mental retardation limited to females [16]. Considering the potential harms, genetic testing should always be performed with the patient's consent or parental consent in the case of minors. A team approach, including a genetic counselor, a psychologist, and a social worker, is recommended throughout the process of evaluation.

In the last years a number of new molecular genetic technologies became available and they promise to change genetic testing for epilepsy, allowing to extend genetic analysis also to sporadic or nonfamilial cases. Two are the major new technologies that can affect the management of epileptic patients: Oligonucleotide Arrays Comparative Genomic Hybridization (Array-CGH) and Next-Generation Sequencing (NGS).

## 2. Molecular Karyotype

During the last 50 years cytogenetics has evolved from simple chromosome counting or banded chromosome morphological identification under light microscope to a molecular approach where chromosomes are analyzed through sophisticated computer system for their ability to hybridize to specific oligonucleotides spanning the entire genome [19]. Array-CGH is nowadays a basic diagnostic tool for clinical diagnosis of several types of developmental delays [20], intellectual disabilities [21, 22], and congenital abnormalities [23]. Epilepsy is also enjoying several advantages from the use of this technology that significantly improves diagnostic resolution of classic cytogenetics [24, 25].

Chromosomes did not become individually identifiable before the discovery that several procedures could create reproducible, permanent, and specific banding patterns [26, 27]. This was fundamental for gene mapping and positional cloning of disease genes and also revealed a large number of rare and subtle pathological conditions that disturbed the normal band patterning of chromosomes. The improvements of high-resolution banding techniques allowed the identification of several subtle chromosomal abnormalities associated with epilepsy [14, 15]. The possibility to study these chromosomes regions with specific hybridization DNA probes through fluorescence in situ hybridization (FISH) greatly improved sensitivity to detect small chromosomal aberrations in specific regions [28].

**Figure 1:**
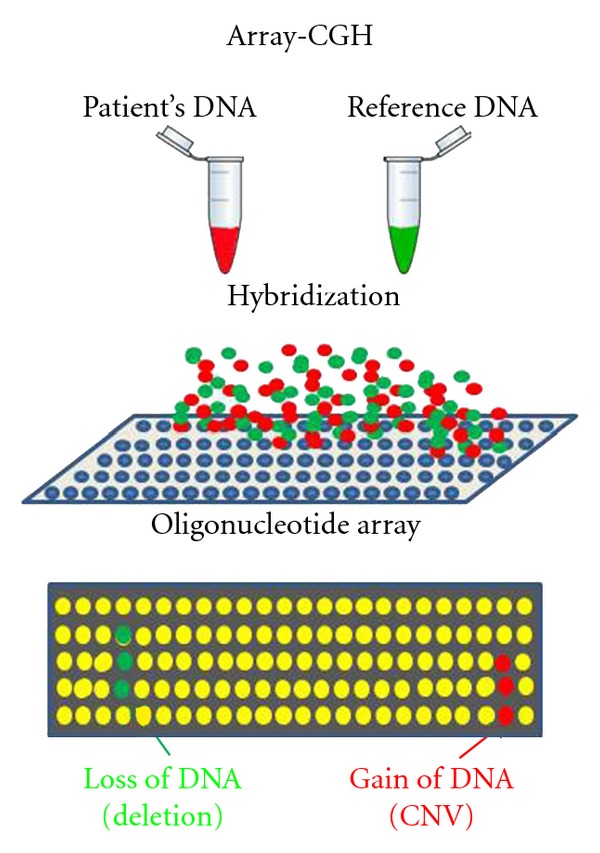


Today molecular karyotyping is rapidly replacing conventional cytogenetics and FISH. This name refers to the analysis of all chromosomes using hybridization to standard DNA sequences arranged on a “chip” rather than microscope observation. The technological development of this approach allows now clinicians to evaluate the entire genome for copy number variants (CNVs, duplications, deletions) in a single test. The high resolution of this approach is however limited by the difficulty to identify balanced chromosome translocations or inversions, even if this powerful technique recognizes in many of them microdeletions or cryptic anomalies at the chromosomal breakpoints. Detection of deletions or duplications is based on the comparison of two genomes (Figure 1). Labelled patient DNA is cohybridized with control DNA to an array spotted with oligonucleotide DNA probes spanning the entire genome at critical intervals. The distance between these oligonucleotide sequences in the genome marks the resolution of the technique and can be as low as 1000 bp. The intensity of the signal from patient and control are then read and normalized by an electronic scanning device coupled with a software that generates a graphic plot of intensities for each probe.

A search of Online Mendelian Inheritance in Man (OMIM) clinical synopsis with the term “seizure” reveals that there are at least 754 mendelian disorders in which epilepsy is or can be part of the clinical condition, but not the main feature. Many of these disorders can be associated with DNA sequence mutations or subtle chromosomal anomalies that can be conveniently detected by array-CGH. Some will be private or sporadic cases and others will be familial. With the widespread use of CGH in both circumstances, many more genetic events will be reported in patients and the genetic aetiology will be recognized making possible over the time to saturate the genome with all possible loci and events that have an epileptogenic role.

## 3. Next-Generation Sequencing

From the publication of the draft of human genome sequence in *Nature*, on 15th February 2001 issue, our view and knowledge of human genome has considerably changed [29] and the technologies to sequence DNA are today of common use in diagnostic practice and much cheaper. Chain termination or Sanger's method [30] was largely used for the Human Genome Project and has dominated the past decades. The logic of this technology was to create by synthesis a population of DNA fragments of different size each one terminated at all possible positions by one of the four labelled dideoxynucleotides (ddNTPs) terminators. Separation of these fragments by polyacrylamide gel or capillary electrophoresis allowed the reading of the sequence through the first developed sequencing machines that could distinguish the fluorescence emitted by the blocking ddNTP [31]. Therefore, these are considered the “first generation” of DNA sequencing technologies.

The need to reduce the cost of large sequencing projects has stimulated the development of a variety of cheaper sequencing technologies that are generally called “Next-Generation Sequencing” (NGS) [32, 33]. The final goal of this new field is to reduce the cost of human genome sequencing till or lower than $1,000 per genome to make it available for common medical practice and diagnostic use [34]. The development of further third-generation sequencing technologies should make possible to sequence single DNA molecules in real time with a cost that it is projected to be very close to the goal [35].

The development of NGS platforms was a major progress in the technology because, differently from Sanger method, rather than producing about one thousand nucleotides for run, they are able to produce orders of magnitude more sequence data using massive parallel process, resulting in substantial increase of data at a lower cost per nucleotide [36, 37].

Several commercial platforms are today available, including Roche/454 [38], Illumina/Solexa and Life Technologies/SOLiD (Table 8(a)). In very general terms these platforms follow similar process that includes: (a) template preparation by breaking large DNA macromolecule to generate short fragment libraries with platform-specific synthetic DNA adapters at the fragment ends, (b) massive and parallel clonal amplification of individual DNA fragment molecules on glass slide or microbeads by PCR [39] to generate a sufficient copy number of the labelled fragment to be detected by the machine optical system, and (c) sequencing by several cycles of extensions that are repeated and detected automatically to create short reads [40]. The data of these reads are then collected by the device, and the alignment of the short reads with specific software allows to rebuild the initial template sequence. Helicos and Pacific Biosystem platforms (Table 8(b)) are substantially different because they use a more advanced laser-based detection system that does not require massive parallel amplification with the considerable advantages to simplify preparation process, to eliminate PCR-induced bias and errors, and to make easy data collection. Ion Torrent developed an entirely new approach to sequencing based on hydrogen ion release when a nucleotide is incorporated into a DNA strand by polymerase (Table 8) [41]. An ion sensor can detect hydrogen ions and convert this ion chemical signal to digital sequence information eliminating the need of optical reading at each dNTP incorporation.

Other third-generation platforms under development make use of nanophotonic visualization chamber, ion semiconductor, electron microscopy, a variety of nanotechnologies like nanopores (Oxford Nanopore Technologies), nanochannels (BioNanomatrix/nanoAnalyzer), nanoparticles (GE Global Research), nanoballs (Complete Genomics), nanowells (CrackerBio), nanoknifes (Renveo), and specially engineered sensor DNA polymerase (VisiGen Biotechnologies) [42]. They promise even larger and faster data production although they are still under development and a few years away from commercial use. In principle also DNA microarrays could allow sequencing by hybridization using ultrafast nonenzymatic methods (Genizon BioSciences) and somebody even suggests that mass spectrometry might be used to determine mass differences between DNA fragments produced by chain termination [43].

The beginning of several individual genome projects has gradually decreased the cost of sequencing an individual genome, and it is likely that the $1,000 cost per person will be reached in few years. In medicine, the “personal genome” age made possible by NGS will be an important milestone for the entire genomic field and will mark a transition from single gene testing to whole genome evaluation [44].

It is impossible to predict today which NGS will eventually dominate genomic research, but it is sure that cost reductions, sequencing speed, and better accuracy will make NGS an essential molecular tool in several areas of biology and medicine.

Although the cost of whole genome sequencing has dropped significantly, it remains a major obstacle since it can reach $100,000 for a single individual. However, targeting sequencing of specific regions of interest can decrease the overall cost and improve efficiency of NGS making this technology ready for diagnostic use [45].

Also the field of epilepsy is potentially affected by NGS. Indeed too many genes and genetic conditions can be associated with epilepsy to make impossible for the clinicians a general use of specific monogene test for the vast majority of nonsyndromic or idiopathic epileptic patients. NGS is changing this situation by targeting several genome regions where known epilepsy genes are located and using enrichment techniques to significantly reduce the cost and improve efficiency. Targeted sequencing usually tests all protein-coding exons (functional exosome) which only requires roughly 5% as much sequencing than whole genome. This strategy will reduces the cost to about $3,000 or even less per single individual. Targeted selection technologies have been marketed and successfully used in different NGS projects and are becoming the tool of choice in several conditions, including epilepsy [46].

## 4. Targeting Sequencing and Epilepsy Gene Panels

A diagnostic panel is the contemporaneous targeted sequencing of a number of known genes that have already been identified as cause of a particular disease. A diagnostic panel is very different from whole genome or exome sequencing. Only genes clearly associated with a disease are examined. The genes included in the panel can be decided by the prescribing physician or by ad hoc committees of experts that can reach a consensus on the number and type of genes to test making commercially available diagnostic panel kits for specific diseases. This strategy should make easier to detect genetic variants that after validation by Sanger sequencing can be interpreted as the cause of the disease. Of course diagnostic panels and targeted sequencing make sense only if the condition is caused by several or very large number of genes. Many genetic disorders fall in this condition and are excellent candidate for the development of diagnostic panels. Epilepsy is an excellent example of such situation since it is a relative frequent disease affecting 1% of the population in a variety of forms, at different ages, with different progression. A genetic cause of epilepsy can be reasonably supposed in sporadic cases if trauma, tumor, or infection can be ruled out. In such circumstance all genetic information about epilepsy genes identified over the years in familial cases can be used to identify the causative gene through an epilepsy diagnostic panel. Indeed in the case of epilepsy the identified genes are so many that they can be classified in subpanels of genes that underline a common clinical entity (Table 9). Clinical considerations may suggest the clinician to include in a diagnostic panel other genes or genes from different sub-panels. At the present the first diagnostic panels for epilepsy that can analyze up to 400 different genes (CeGaT GmbH) are commercially available [47].

## 5. Future Perspectives and Conclusions

The genetics of epilepsy has evolved from ion channel and neurotransmitter receptor subunits to newly discovered genes highlighting the importance of different pathways in the epileptogenesis. Furthermore, it has been demonstrated that copy number variations collectively explain a larger portion of idiopathic epilepsy than any single gene. These studies have identified structural genomic variations associated with idiopathic epilepsy, representing a change from the conventional knowledge that chromosome microarray analysis is useful only for patients with intellectual disability or dysmorphism [48]. Genetic testing techniques are rapidly evolving and whole exome or whole genome sequencing, performing at increasingly cheaper costs, will allow rapid discovery of other pathogenic mutations, variants in noncoding DNA, and copy number variations encompassing several genes. This rapidly accumulating genetic information will expand our understanding of epilepsy, and will allow more rational and effective treatment. However, along with the ability to identify genetic variants potentially associated with epilepsy it is imperative to validate genetic associations and analyze their clinical significance.

Is it worth? The main objective of a diagnostic panel for epilepsy is to discover the molecular defect in all possible cases to create a specific and personalized treatment of the disease than can be pharmacologically different for different types of molecular defects. Personalized therapy will be possible only within a genomic medicine. But genomic medicine at the same time will raise other questions: what to do if more than one genetic variant is identified in the same epileptic patient? Can we understand how genetic interactions will modulate the disease severity and prognosis? Can interaction of specific genetic variants and environmental factors modulate the clinical spectrum of the disorder? What to do if the diagnostic panel is inconclusive? Are the costs affordable? These are only few questions that the genomics of epilepsy will raise. The answers will require time, a lot of sequencing, and probably the development of new and cheaper sequencing technologies.

## Figures and Tables

**Table 1:** Disease genes identified in generalized myoclonic epilepsy, febrile seizures, absences (37 genes).

Gene Symbol	Gene name and description
ALDH7A1	Aldehyde dehydrogenase 7 family, member A1
BRD2	Bromodomain containing 2
CACNA1A	Calcium channel, voltage-dependent, P/Q type, alpha 1A subunit
CACNA1H	Calcium channel, voltage-dependent, T type, alpha 1H subunit
CACNB4	Calcium channel, voltage-dependent, beta 4 subunit
CASR	Calcium-sensing receptor
CHRNA2	Cholinergic receptor, nicotinic, alpha 2 (neuronal)
CHRNA4	Cholinergic receptor, nicotinic, alpha 4
CHRNB2	Cholinergic receptor, nicotinic, beta 2 (neuronal)
CLCN2	Chloride channel 2
CSTB	Cystatin B (stefin B)
EFHC1	EF-hand domain (C-terminal) containing 1
EPM2A	Epilepsy, progressive myoclonus type 2A, Lafora disease (laforin)
GABRA1	Gamma-aminobutyric acid (GABA) A receptor, alpha 1
GABRB3	Gamma-aminobutyric acid (GABA) A receptor, beta 3
GABRD	Gamma-aminobutyric acid (GABA) A receptor, delta
GABRG2	Gamma-aminobutyric acid (GABA) A receptor, gamma 2
GPR98	G protein-coupled receptor 98
GRIN2A	Glutamate receptor, ionotropic, N-methyl D-aspartate 2A
GRIN2B	Glutamate receptor, ionotropic, N-methyl D-aspartate 2B
KCNMA1	Potassium large conductance calcium-activated channel, subfamily M, alpha member 1
KCNQ2	Potassium voltage-gated channel, KQT-like subfamily, member 2
KCNQ3	Potassium voltage-gated channel, KQT-like subfamily, member 3
KCTD7	Potassium channel tetramerisation domain containing 7
MBD5	Methyl-CpG-binding domain protein 5
ME2	Malic enzyme 2, NAD(+)-dependent, mitochondrial
NHLRC1	NHL repeat containing 1
PCDH19	Protocadherin 19
PRICKLE1	Prickle homolog 1 (Drosophila)
PRICKLE2	Prickle homolog 2 (Drosophila)
SCARB2	Scavenger receptor class B, member 2
SCN1A	Sodium channel, voltage-gated, type I, alpha subunit
SCN1B	Sodium channel, voltage-gated, type I, beta subunit
SCN2A	Sodium channel, voltage-gated, type II, alpha subunit
SCN9A	Sodium channel, voltage-gated, type IX, alpha subunit
SLC2A1	Solute carrier family 2 (facilitated glucose transporter), member 1
TBC1D24	TBC1 domain family, member 24

**Table 2:** Disease genes identified in syndromic epilepsy (47 genes).

Gene symbol	Gene name and description	Syndrome
ARFGEF2	ADP-ribosylation factor GEF2	Periventricular heterotopia
ARHGEF9	Cdc42 GEF 9	Hyperekplexia with epilepsy
A2BP1	Ataxin 2-binding protein 1 (RNA binding protein fox-1 homolog 1)	Mental retardation and epilepsy
ASPA	Aspartoacylase	Canavan syndrome
ATP1A2	ATPase, Na/K transporting, alpha 2 polypeptide	Familial hemiplegic migraine
ATP2A2	ATPase, Ca transporting, cardiac muscle, slow twitch 2	Darier-White syndrome
ATP6V0A2	ATPase, H+ transporting, lysosomal V0 subunit a2	Cutis laxa with epilepsy and mental retardation
CACNA1A	Calcium channel, voltage-dependent, P/Q type, alpha 1A subunit	Familial hemiplegic migraine
CCDC88C	Coiled-coil domain containing 88C	Hydrocephalus with medial diverticulum
CLCNKA	Chloride channel Ka	Bartter syndrome
CLCNKB	Chloride channel Kb	Bartter syndrome
COH1	Cohen syndrome protein 1—vacuolar protein sorting 13 homolog B	Cohen syndrome
DLGAP2	Discs, large (Drosophila) homolog-associated protein 2	Progressive epilepsy with mental retardation
GFAP	Glial fibrillary acidic protein	Alexander disease
GLI3	GLI family zinc finger 3	Pallister-hall syndrome
GLRA1	Glycine receptor, alpha 1	Hyperekplexia
GLRB	Glycine receptor, beta	Hyperekplexia
GPHN	Gephyrin	Hyperekplexia
KCNA1	Potassium voltage-gated channel, shaker-related	Episodic ataxia
KCNJ1	Potassium inwardly rectifying channel, subfamily J, member 1	Bartter syndrome
KCNJ10	Potassium inwardly rectifying channel, subfamily J, member 10	Seizures, deafness, ataxia, mental retardation
KIAA1279	Kinesin family member 1 binding protein	Goldberg-Shprintzen
LAMA2	Laminin, alpha 2	Merosin deficiency
LBR	Lamin B receptor	Pelger-Huet syndrome
LGI1	Leucine-rich, glioma inactivated 1	Autosomal dominant lateral temporal lobe epilepsy
MLC1	Megalencephalic leukoencephalopathy with subcortical cysts 1	Megalencephalic leukoencephalopathy with cysts
MLL2	Myeloid/lymphoid or mixed-lineage leukemia 2	Kabuki syndrome
NF1	Neurofibromin 1	Neurofibromatosis
NIPBL	Nipped-B homolog (Drosophila)	Cornelia de Lange syndrome
PANK2	Pantothenate kinase 2	Neurodegeneration with brain iron accumulation
PI12	Serpin peptidase inhibitor, clade I (neuroserpin), member 1	Encephalopathy with neuroserpin inclusion bodies
PIGV	Phosphatidylinositol glycan anchor biosynthesis, class V	Hyperphosphatasia with mental retardation
PLA2G6	Phospholipase A2, group VI (cytosolic, calcium independent)	Infantile neuroaxonal dystrophy
RAI1	Retinoic acid induced 1	Smith Magenis syndrome
SCN8A	Sodium channel, voltage gated, type VIII, alpha subunit	Cerebellar atrophy, ataxia, and mental retardation
SETBP1	SET binding protein 1	Schinzel-Giedion midface retraction syndrome
SHH	Sonic hedgehog	Holoprosencephaly
SLC4A10	Solute carrier family 4, sodium bicarbonate transporter, member 10	Epilepsy with mental retardation
SLC6A5	Solute carrier family 6 (neurotransmitter transporter, glycine), member 5	Hyperekplexia
SMC1A	Structural maintenance of chromosomes 1A	Cornelia de lange syndrome
SMC3	Structural maintenance of chromosomes 3	Cornelia de lange syndrome
SYNGAP1	Synaptic Ras GTPase activating protein 1	Epilepsy and mental retardation
TBX1	T-box 1	Di George syndrome
TSC1	Tuberous sclerosis 1	Tuberous sclerosis
TSC2	Tuberous sclerosis 2	Tuberous sclerosis
VPS13A	Vacuolar protein sorting 13 homolog A	Neuroacanthocytosis
ZEB2	Zinc finger E-box binding homeobox 2	Mowat-Wilson syndrome

**Table 3:** Disease genes identified in epileptic encephalopathies (30 genes).

Gene symbol	Gene Name and Description	Diseases
ARHGEF9	Cdc42 guanine nucleotide exchange factor (GEF) 9	Early infantile epileptic encephalopathy
ARX	Aristaless related homeobox	Early infantile epileptic encephalopathy
CDKL5	Cyclin-dependent kinase-like 5	Early infantile epileptic encephalopathy
CNTNAP2	Contactin associated protein-like 2	Pitt Hopkins syndrome
FOXG1	Forkhead box G1	Rett syndrome
GABRG2	Gamma-aminobutyric acid (GABA) A receptor, gamma 2	Early infantile epileptic encephalopathy
GRIN2A	Glutamate receptor, ionotropic, N-methyl D-aspartate 2A	Early infantile epileptic encephalopathy
GRIN2B	Glutamate receptor, ionotropic, N-methyl D-aspartate 2B	Early infantile epileptic encephalopathy
MAPK10	Mitogen-activated protein kinase 10	Lennox Gastaut syndrome
MECP2	Methyl CpG binding protein 2	Rett syndrome
NRXN1	Neurexin 1	Pitt Hopkins Syndrome
PCDH19	Protocadherin 19	Early infantile epileptic encephalopathy
PNKP	Polynucleotide kinase 3'-phosphatase	Early infantile epileptic encephalopathy
RNASEH2A	Ribonuclease H2, subunit A	Aicardi-Goutieres syndrome
RNASEH2B	Ribonuclease H2, subunit B	Aicardi-Goutieres syndrome
RNASEH2C	Ribonuclease H2, subunit C	Aicardi-Goutieres syndrome
SAMHD1	SAM domain and HD domain 1	Aicardi-Goutieres syndrome
SCN1A	Sodium channel, voltage-gated, type I, alpha subunit	Early infantile epileptic encephalopathy
SCN1B	Sodium channel, voltage-gated, type I, beta subunit	Early Infantile epileptic encephalopathy
SCN2A	Sodium channel, voltage-gated, type II, alpha subunit	Early infantile epileptic Encephalopathy
SCN9A	Sodium channel, voltage-gated, type IX, alpha subunit	Early infantile epileptic encephalopathy
SLC2A1	Solute carrier family 2 (facilitated glucose transporter), member 1	GLUT1 deficiency syndrome
SLC25A22	Solute carrier family 25 (mitochondrial carrier: glutamate), member 22	Early infantile epileptic encephalopathy
SLC9A6	Solute carrier family 9 (sodium/hydrogen exchanger), member 6	Angelman syndrome
SPTAN1	Spectrin, alpha, non-erythrocytic 1 (alpha-fodrin)	Early infantile epileptic encephalopathy
STXBP1	Syntaxin binding protein 1	Early infantile epileptic encephalopathy
TCF4	Transcription factor 4	Pitt Hopkins syndrome
TREX1	Three prime repair exonuclease 1	Aicardi-Goutieres syndrome
UBE3A	Ubiquitin protein ligase E3A	Angelman syndrome
ZEB2	Zinc finger E-box binding homeobox 2	Mowat-Wilson syndrome

**Table 4:** Epilepsy with mental retardation and brain malformations.

Gene symbol	Name	Disease
	(a) Mental retardation (25 genes)	

ARHGEF9	Cdc42 guanine nucleotide exchange factor (GEF) 9	Early infantile epileptic encephalopathy
ARX	Aristaless related homeobox	Early infantile epileptic encephalopathy
ATP6AP2	ATPase, H+ transporting, lysosomal accessory protein 2	Epilepsy with XLMR*
ATRX	Alpha thalassemia/mental retardation syndrome X-linked	Epilepsy with XLMR*
CASK	Calcium/calmodulin-dependent serine protein kinase (MAGUK family)	Mental retardation and microcephaly
CDKL5	Cyclin-dependent kinase-like 5	Early infantile epileptic encephalopathy
CUL4B	Cullin 4B	Epilepsy with XLMR*
CXORF5	Oral-facial-digital syndrome 1	Simpson-Golabi-Behmel syndrome
DCX	Doublecortin	Lissencephaly
FGD1	FYVE, RhoGEF and PH domain containing 1	Aarskog-Scott syndrome
GPC3	Glypican 3	Simpson-Golabi-Behmel syndrome
GRIA3	Glutamate receptor, ionotrophic, AMPA 3	Epilepsy with XLMR*
HSD17B10	Hydroxysteroid (17-beta) dehydrogenase 10	Epilepsy with XLMR*
JARID1C	Lysine (K)-specific demethylase 5C	Epilepsy with XLMR*
OPHN1	Oligophrenin 1	Epilepsy with XLMR*
PAK3	P21 protein (Cdc42/Rac)-activated kinase 3	Epilepsy with XLMR*
PHF6	PHD finger protein 6	Borjeson Forssmann Lehmann syndrome
PLP1	Proteolipid protein 1	Pelizaeus-Merzbacher disease
PQBP1	Polyglutamine binding protein 1	Epilepsy with XLMR*
RAB39B	RAB39B, member RAS oncogene family	Epilepsy with XLMR*
SLC9A6	Solute carrier family 9 (sodium/hydrogen exchanger), member 6	Angelman-Like syndrome
SMC1A	Structural maintenance of chromosomes 1A	Cornelia De Lange syndrome
SMS	Spermine synthase	Epilepsy with XLMR*
SRPX2	Sushi-repeat containing protein, X-linked 2	Rolandic epilepsy
SYP	Synaptophysin	Epilepsy with XLMR*
		*XLMR: X-linked mental retardation

	(b) Joubert syndrome (10 genes)	

AHI1	Abelson helper integration site 1	Joubert syndrome
ARL13B	ADP-ribosylation factor-like 13B	Joubert syndrome
CC2D2A	Coiled-coil and C2 domain containing 2A	Joubert syndrome
CEP290	Centrosomal protein 290 kDa	Joubert syndrome
CXORF5	Oral-facial-digital syndrome 1	Joubert syndrome
INPP5E	Inositol polyphosphate-5-phosphatase, 72 kDa	Joubert syndrome
NPHP1	Nephronophthisis 1 (juvenile)	Joubert syndrome
RPGRIP1L	Retinitis pigmentosa GTPase regulator interacting protein 1 like	Joubert syndrome
TMEM67	Transmembrane protein 67	Joubert syndrome
TMEM216	Transmembrane protein 216	Joubert syndrome

	(c) Lissencephaly and polymicrogyria (18 genes)	

COL18A1	Collagen, type XVIII, alpha 1	Polymicrogyria
CPT2	Carnitine palmitoyltransferase 2	Polymicrogyria
DCX	Doublecortin	Lissencephaly
EOMES	Eomesodermin	Polymicrogyria
FGFR3	Fibroblast growth factor receptor 3	Polymicrogyria
FLNA	Filamin A, alpha	Periventricular heterotopia
GPR56	G protein-coupled receptor 56	Polymicrogyria
PAFAH1B1	Platelet-activating factor acetylhydrolase 1b, regulatory subunit 1 (45kDa)	Lissencephaly
PAX6	Paired box 6	Polymicrogyria
PEX7	Peroxisomal biogenesis factor 7	Polymicrogyria
RAB3GAP1	RAB3 GTPase activating protein subunit 1 (catalytic)	Warburg microsyndrome
RELN	Reelin	Lissencephaly
SNAP29	Synaptosomal-associated protein, 29 kDa	Cerebral dysgenesis
SRPX2	Sushi-repeat containing protein, X-linked 2	Rolandic epilepsy
TUBA1A	Tubulin, alpha 1a	Lissencephaly
TUBA8	Tubulin, alpha 8	Polymicrogyria
TUBB2B	Tubulin, beta 2B	Polymicrogyria
VDAC1	Voltage-dependent anion channel 1	Polymicrogyria

	(d) Severe microcephaly and pontocerebellar hypoplasia (22 genes)	

ASPM	Asp (abnormal spindle) homolog, microcephaly associated (Drosophila)	Microcephaly
ATR	Ataxia telangiectasia and Rad3 related	Microcephaly
BUB1B	Budding uninhibited by benzimidazoles 1 homolog beta (yeast)	Microcephaly
CASK	Calcium/calmodulin-dependent serine protein kinase (MAGUK family)	Microcephaly
CDK5RAP2 [Microcephaly]	CDK5 regulatory subunit associated protein 2	Microcephaly
CENPJ	Centromere protein J	Microcephaly
CEP152	Centrosomal protein 152 kDa	Microcephaly
LIG4	Ligase IV, DNA, ATP-dependent	Microcephaly
MCPH1	Microcephalin 1	Microcephaly
MED17	Mediator complex subunit 17	Microcephaly
NHEJ1	Nonhomologous end-joining factor 1	Microcephaly
PCNT	Pericentrin	Microcephalic osteodysplastic Dwarfism
PNKP	Polynucleotide kinase 3^'^-phosphatase	Microcephaly
PQBP1	Polyglutamine binding protein 1	X-linked mental retardation
RARS2	Arginyl-tRNA synthetase 2, mitochondrial	Pontocerebellar hypoplasia
SLC25A19	Solute carrier family 25 (mitochondrial thiamine pyrophosphate carrier), member 19	Microcephaly
STIL	SCL/TAL1 interrupting locus	Microcephaly
TSEN2	tRNA splicing endonuclease 2 homolog (S. cerevisiae)	Pontocerebellar hypoplasia
TSEN34 [Pontocerebellar Hypoplasia]	tRNA splicing endonuclease 34 homolog (S. cerevisiae)	Pontocerebellar hypoplasia
TSEN54 [Pontocerebellar Hypoplasia]	tRNA splicing endonuclease 54 homolog (S. cerevisiae)	Pontocerebellar hypoplasia
VRK1	Vaccinia related kinase 1	Pontocerebellar hypoplasia
WDR62	WD repeat domain 62	Microcephaly, cortical malformations and mental retardation

	(e) Walker-Warburg syndrome (WWS) or muscle, eye and brain disease (6 genes) anomalies type A2 (MDDGA2)	

FKRP	Fukutin-related protein	Walker-Warburg syndrome
FKTN	Fukutin	Walker-Warburg syndrome
LARGE	Like-glycosyltransferase	Walker-Warburg syndrome
POMGNT1	Protein O-linked mannose beta1,2-N-acetylglucosaminyltransferase	Walker-Warburg syndrome
POMT1	Protein-O-mannosyltransferase 1	Walker-Warburg syndrome
POMT2	Protein-O-mannosyltransferase 2	Walker-Warburg Syndrome

	(f) Holoprosencephaly (HPE) (8 genes)	

FGF8	Fibroblast growth factor 8 (androgen-induced)	Holoprosencephaly
GLI2	GLI family zinc finger 2	Holoprosencephaly 9
GLI3	GLI family zinc finger 3	Greig cephalopolysyndactyly syndrome
PTCH1	patched 1	Holoprosencephaly 7
SHH	Sonic Hedgehog	Holoprosencephaly 3
SIX3	SIX homeobox 3	Holoprosencephaly 2
TGIF1	TGFB-induced factor homeobox 1	Holoprosencephaly 4
ZIC2	Zic family member 2	Holoprosencephaly 5

**Table 5:** Epilepsy with other neurological problems.

Gene symbol	Name	Disease
	(a) Leukodystrophies (20 genes)	

ARSA	Arylsulfatase A	Leukodystrophy metachromatic (MLD)
ASPA	Aspartoacylase	Canavan disease
EIF2B1	Eukaryotic translation initiation factor 2B, subunit 1 alpha, 26 kDa	Leukodystrophy
EIF2B2	Eukaryotic translation initiation factor 2B, subunit 2 beta, 39 kDa	Leukodystrophy
EIF2B3	Eukaryotic translation initiation factor 2B, subunit 3 gamma, 58 kDa	Leukodystrophy
EIF2B4	Eukaryotic translation initiation factor 2B, subunit 4 delta, 67 kDa	Leukodystrophy
EIF2B5	Eukaryotic translation initiation factor 2B, subunit 5 epsilon, 82 kDa	Leukodystrophy
GALC	Galactosylceramidase	Leukodystrophy globoid cell (GLD)
GFAP	Glial fibrillary acidic protein	Alexander disease
MLC1	Megalencephalic leukoencephalopathy with subcortical cysts 1	Megalencephalic leukoencephalopathy
NOTCH3	Notch3	CADASIL
PLP1	Proteolipid protein 1	Leukodystrophy hypomyelinating type 1 (HLD1)
PSAP	Prosaposin	Leukodystrophy metachromatic
RNASEH2A	Ribonuclease H2, subunit A	Aicardi-Goutieres syndrome type 4 (AGS4)
RNASEH2B	Ribonuclease H2, subunit B	Aicardi-Goutieres syndrome type 2 (AGS2)
RNASEH2C	Ribonuclease H2, subunit C	Aicardi-Goutieres syndrome type 3 (AGS3
SAMHD1	SAM domain and HD domain 1	Aicardi-Goutieres syndrome type 5 (AGS5)
SDHA	Succinate dehydrogenase complex, subunit A, flavoprotein (Fp)	Leigh syndrome
SUMF1	Sulfatase modifying factor 1	Multiple sulfatase deficiency (MSD)
TREX1	Three prime repair exonuclease 1	Aicardi-Goutieres syndrome type 1 (AGS1)

	(b) Migraine (6 genes)	

ATP1A2	ATPase, Na+/K+ transporting, alpha 2 polypeptide	Migraine familial hemiplegic type 2 (FHM2)
CACNA1A	Calcium channel, voltage-dependent, P/Q type, alpha 1A subunit	Spinocerebellar ataxia type 6 (SCA6)
NOTCH3	Notch 3	CADASIL
POLG	Polymerase (DNA directed), gamma	Progressive external ophthalmoplegia
SCN1A	Sodium channel, voltage-gated, type I, alpha subunit	Migraine familial hemiplegic type 3 (FHM3)
SLC2A1	Solute carrier family 2 (facilitated glucose transporter), member 1	GLUT1 deficiency type 1 (GLUT1DS1) syndrome

	(c) Disorders of Ras-MAPK pathway with epilepsy (13 genes)	

BRAF	V-raf murine sarcoma viral oncogene homolog B1	Cardiofaciocutaneous (CFC ) syndrome
CBL	Cas-Br-M (murine) ecotropic retroviral transforming sequence	Noonan syndrome-like disorder (NSL)
HRAS	V-Ha-ras Harvey rat sarcoma viral oncogene homolog	Faciocutaneoskeletal (FCSS) syndrome
KRAS	V-Ki-ras2 Kirsten rat sarcoma viral oncogene homolog	Noonan type 3 (NS3) syndrome
MAP2K1	Mitogen-activated protein kinase kinase 1	cardiofaciocutaneous (CFC) syndrome
MAP2K2	Mitogen-activated protein kinase kinase 2	cardiofaciocutaneous (CFC) syndrome
NF1	Neurofibromin 1	Neurofibromatosis type 1
NRAS	Neuroblastoma RAS viral (v-ras) oncogene homolog	Noonan type 6 (NS6) syndrome
PTPN11	Protein tyrosine phosphatase, non-receptor type 11	LEOPARD type 1 (LEOPARD1) syndrome
RAF1	V-raf-1 murine leukemia viral oncogene homolog 1	Noonan type 5 (NS5) syndrome
SHOC2	Soc-2 suppressor of clear homolog (C. elegans)	Noonan syndrome-like with loose anagen hair
SOS1	Son of sevenless homolog 1 (Drosophila)	Noonan type 4 (NS4) syndrome
SPRED1	Sprouty-related, EVH1 domain containing 1	Neurofibromatosis type 1-like syndrome

	(d) Hyperekplexia (5 genes)	

ARHGEF9	Cdc42 guanine nucleotide exchange factor (GEF) 9	Hyperekplexia with epilepsy
GLRA1	Glycine receptor, alpha 1	Hyperekplexia with epilepsy
GLRB	Glycine receptor, beta	Hyperekplexia with epilepsy
GPHN	Gephyrin	Hyperekplexia with epilepsy
SLC6A5	solute carrier family 6 (neurotransmitter, transporter, glycine), member 5	Hyperekplexia with epilepsy

	(e) Neuronal migration disorders (31 genes)	

ARFGEF2	ADP-ribosylation factor guanine nucleotide-exchange factor 2 (brefeldin A-inhibited)	Microcephaly
ARX	Aristaless-related homeobox	Early infantile epileptic encephalopathy
COL18A1	Collagen, type XVIII, alpha 1	Polymicrogyria
COL4A1	Collagen, type IV, alpha 1	Porencephaly
CPT2	Carnitine palmitoyltransferase 2	Polymicrogyria
DCX	Doublecortin	Lissencephaly
EMX2	Empty spiracles homeobox 2	Schizencephaly
EOMES	Eomesodermin	Polymicrogyria
FGFR3	Fibroblast growth factor receptor 3	Polymicrogyria
FKRP	Fukutin related protein	Walker-Warburg syndrome
FKTN	Fukutin	Walker-Warburg syndrome
FLNA	Filamin A, alpha	Periventricular heterotopia
GPR56	G protein-coupled receptor 56	Polymicrogyria
LAMA2	Laminin, alpha2	Merosin deficiency
LARGE	Like-glycosyltransferase	Walker-Warburg syndrome
PAFAH1B1	Platelet-activating factor acetylhydrolase 1b, regulatory subunit 1 (45 kDa)	Lissencephaly
PAX6	Paired box 6	Polymicrogyria
PEX7	Peroxisomal biogenesis factor 7	Polymicrogyria
POMGNT1	Protein O-linked mannose beta1,2-N-acetylglucosaminyltransferase	Walker-Warburg syndrome
POMT1	Protein O-mannosyltransferase 1	Walker-Warburg syndrome
POMT2	Protein O-mannosyltransferase 2	Walker-Warburg syndrome
PQBP1	Polyglutamine binding protein 1	X-linked mental retardation
RAB3GAP	RAB3 GTPase activating protein subunit 1 (catalytic)	Warburg microsyndrome
RELN	Reelin	Lissencephaly
SNAP29	Synaptosomal-associated protein, 29 kDa	Cerebral dysgenesis
SRPX2	Sushi-repeat containing protein, X-linked 2	Rolandic epilepsy
TUBA1A	Tubulin, alpha 1a	Lissencephaly
TUBA8	Tubulin, alpha 8	Polymicrogyria
TUBB2B	Voltage-dependent anion channel 1	Polymicrogyria
VDAC1	Voltage-dependent anion channel 1	Polymicrogyria
WDR62	WD repeat domain 62	Microcephaly, cortical malfor, mental retardatation

**Table 6:** Inherited errors of metabolism with epilepsy (49 genes).

Gene symbol	Defective enzyme name	Disease
ABCC8	ATP-binding cassette, subfamily C (CFTR/MRP), member 8	Hypoglcemia
ACY1	Aminoacylase1	Aminoacylase1 deficiency
ADSL	Adenylosuccinate lyase	Adenylosuccinase deficiency
AGA	Aspartylglucosaminidase	Aspartylglucosaminuria
ALDH4A1	Aldehyde dehydrogenase 4 family, member A1	Hyperprolinemia
ALDH5A1	Aldehyde dehydrogenase 5 family, member A1	Succinic Semialdehyde dehydrogenase deficiency
ALDH7A1	Aldehyde dehydrogenase 7 family, member A1	Pyridoxine deficiency
ARG1	Liver arginase	Argininemia
ARSA	Arylsulfatase A	Metachromatic leukoodystrophy
ASPA	Aspartoacylase	Canavan disease
ATIC	5-aminoimidazole-4-carboxamide ribonucleotide (AICAr) formyltransferase/IMP cyclohydrolase	AICAr transformylase/IMP cyclohydrolase deficiency (ATIC Deficiency)
BTD	Biotinidase	Biotinidase deficiency
CPT2	Carnitine palmitoyltransferase 2	Carnitine palmitoyltransferase II deficiency
CTSA	Cathepsin A	Galactosialidosis
DPYD	Dihydropyrimidine dehydrogenase	Dihydropyrimidine dehydrogenase deficiency
ETFA	Electron-transfer-flavoprotein, alpha polypeptide	Glutaraciduria
ETFB	Electron-transfer-flavoprotein, beta polypeptide	Glutaraciduria
ETFDH	Electron-transferring-flavoprotein dehydrogenase	Glutaraciduria
FH	Fumarate hydratase	Fumarase deficiency
FOLR1	Folate receptor 1 (adult)	Cerebral folate transport deficiency
FUCA1	Fucosidase, alpha-L- 1, tissue	Fucosidosis
GALC	Galactosylceramidase	Krabbe disease
GAMT	Guanidinoacetate N-methyltransferase	Guanidinoacetate N-methyltransferase deficiency
GCDH	Glutaryl-CoA dehydrogenase	Glutaraciduria
GCSH	Glycine cleavage system protein H (aminomethyl carrier)	Glycine encephalopathy
GCST	Glycine cleavage system protein T (aminomethyltransferase)	Glycine encephalopathy
GLB1	Galactosidase, beta 1	Gangliosidosis
GLDC	Glycine dehydrogenase (decarboxylating)	Glycine encephalopathy
GNE	Glucosamine (UDP-N-acetyl)-2-epimerase/N-acetylmannosamine kinase	Sialuria
HEXA	Hexosaminidase A (alpha polypeptide)	Gangliosidosis
HEXB	Hexosaminidase B (beta polypeptide)	Gangliosidosis
HPD	4-Hydroxyphenylpyruvate dioxygenase	Tyrosinemia
L2HGDH	L-2-Hydroxyglutarate dehydrogenase	L-2-Hydroxyglutaric aciduria
LAMA2	Laminin, alpha 2	Muscular dystrophy
MOCS1	Molybdenum cofactor synthesis 1	Molybdene cofactor deficiency
MOCS2	Molybdenum cofactor synthesis 2	Molybdene cofactor deficiency
NEU1	Sialidase 1 (lysosomal sialidase)	Neuraminidase deficiency
NPC1	Niemann-Pick disease, type C1	Niemann-Pick disease
NPC2	Niemann-Pick disease, type C2	Niemann-Pick disease
PGK1	Phosphoglycerate kinase 1	GAMT deficiency
PRODH	Proline dehydrogenase (oxidase) 1	Hyperprolinemia
PSAP	Prosaposin	Krabbe disease
QDPR	Quinoid dihydropteridine reductase	Hyperphenylalaninemia
SLC17A5	Solute carrier family 17 (anion/sugar transporter), member 5	Sialuria
SLC25A15	Solute carrier family 25 (mitochondrial carrier; ornithine transporter) member 15	Ornithine translocase deficiency
SLC46A1	Solute carrier family 46 (folate transporter), member 1	Folate malabsorption
SMPD1	Sphingomyelin phosphodiesterase 1, acid lysosomal	Niemann pick disease
SUMF1	Sulfatase modifying factor 1	Sulfatidosis
SUOX	Sulfite oxidase	Sulfitoxidasis

**Table 7:** Other inherited errors of metabolism with epilepsy.

Gene symbol	Defective enzyme name	Disease
	(a) Congenital Disorder of Glycosylation (CDG) (23 genes)	

ALG1	N-linked glycosylation 1, beta-1,4-mannosyltransferase homolog	CDG
ALG2	N-linked glycosylation 2, alpha-1,3-mannosyltransferase homolog	CDG
ALG3	N-linked glycosylation 3, alpha-1,3-mannosyltransferase homolog	CDG
ALG6	N-linked glycosylation 6, alpha-1,3-glucosyltransferase homolog	CDG
ALG8	N-linked glycosylation 8, alpha-1,3-glucosyltransferase homolog	CDG
ALG9	N-linked glycosylation 9, alpha-1,3-glucosyltransferase homolog	CDG
ALG12	N-linked glycosylation 12, alpha-1,3-glucosyltransferase homolog	CDG
B4GALT1	UDP-Gal: betaGlcNAc beta 1,4-galactosyltransferase, polypeptide 1	CDG
COG1	Component of oligomeric golgi complex 1	CDG
COG7	Component of oligomeric golgi complex 7	CDG
COG8	Component of oligomeric golgi complex 8	CDG
DOLK	Dolichol kinase	CDG
DPAGT1	Dolichyl-phosphate (UDP-N-acetylglucosamine) N-acetyl glucosamine phosphotransferase 1 (GlcNAc-1-P transferase)	CDG
DPM1	Dolichyl-phosphate mannosyltransferase polypeptide 1, catalytic subunit	CDG
DPM3	Dolichyl-phosphate mannosyltransferase polypeptide 3	CDG
MOGS	Mannosyl-oligosaccharide glucosidase	CDG
MGAT2	Mannosyl (alpha-1,6-)-glycoprotein beta-1,2-N-acetylglucosaminyltransferase	CDG
MPDU1	Mannose-P-dolichol utilization defect 1	CDG
MPI	Mannose phosphate isomerase	CDG
PMM2	Phosphomannomutase 2	CDG
RFT1	Requiring fifty three 1 homolog	CDG
SLC35A1	Solute carrier family 35 (CMP-sialic acid transporter), member A1	CDG
SLC35C1	Solute carrier family 35, member C1	CDG

	(b) Neuronal ceroid lipofuscinosis (NCL) (8 genes)	

CLN3	Ceroid-lipofuscinosis, neuronal 3	NLC
CLN5	Ceroid-lipofuscinosis, neuronal 5	NLC
CLN6	Ceroid-lipofuscinosis, neuronal 6	NLC
CLN8	Ceroid-lipofuscinosis, neuronal 8	NLC
CTSD	Cathepsin D	NLC
MFSD8	Major facilitator superfamily domain containing 8	NLC
PPT1	Palmitoyl-protein thioesterase 1	NLC
TPP1	Tripeptidyl peptidase I	NLC

	(c) Defects of mitochondrial metabolism including coenzyme Q deficiency (35 genes)	

APTX	Aprataxin	Coenzyme Q10 Deficiency
ATPAF2	ATP synthase mitochondrial F1 complex assembly factor 2	ATPase deficiency
BCS1L	BCS1-like	Leigh syndrome
C12ORF65	Chromosome 12 open reading frame 65	Leigh syndrome
C8ORF38	Chromosome 8 open reading frame 38	Leigh syndrome
CABC1	Chaperone activity of bc1 complex-like, mitochondria	Coenzyme Q10 deficiency
COQ2	Coenzyme Q2 homolog, prenyltransferase (yeast)	Coenzyme Q10 deficiency
COQ9	Coenzyme Q9 homolog (S. cerevisiae)	Coenzyme Q10 deficiency
COX10	COX10 homolog, cytochrome c oxidase assembly protein, heme A: farnesyltransferase (yeast)	Leigh syndromeCOX10
COX15	COX15 homolog, cytochrome c oxidase assembly protein (yeast)	Leigh syndrome
DLD	Dihydrolipoamide dehydrogenase	Leigh syndrome
GCSH	Glycine cleavage system protein H (aminomethyl carrier)	Glycine encephalopathy
GCST	Aminomethyltransferase (glycine cleavage system protein T)	Glycine encephalopathy
GLDC	Glycine dehydrogenase (decarboxylating)	Glycine encephalopathy
HSD17B10	Hydroxysteroid (17-beta) dehydrogenase 10	HSD17B10 deficiency
LRPPRC	Leucine-rich PPR-motif containing	Leigh syndrome
NDUFA2	NADH dehydrogenase (ubiquinone) 1 alpha subcomplex, 2,8 kDa	Leigh syndrome
NDUFS1	NADH dehydrogenase (ubiquinone) Fe-S protein 1, 75 kDa	Leigh syndrome
NDUFS3	NADH dehydrogenase (ubiquinone) Fe-S protein 3, 30 kDa	Leigh syndrome
NDUFS4	NADH dehydrogenase (ubiquinone) Fe-S protein 4, 18 kDa	Leigh syndrome
NDUFS7	NADH dehydrogenase (ubiquinone) Fe-S protein 7, 20 kDa	Leigh syndrome
NDUFS8	NADH dehydrogenase (ubiquinone) Fe-S protein 8, 23 kDa	Leigh syndrome
NDUFV1	NADH dehydrogenase (ubiquinone) flavoprotein 1, 51 kDa	Leigh syndrome
PC	Pyruvate carboxylase	Leigh syndrome
PDHA1	Pyruvate dehydrogenase (lipoamide) alpha 1	Leigh syndrome
PDSS1	Prenyl (decaprenyl) diphosphate synthase, subunit 1	Coenzyme Q10 deficiency
PDSS2	Prenyl (decaprenyl) diphosphate synthase, subunit 2	Coenzyme Q10 deficiency]
POLG	Polymerase (DNA directed), gamma	Mitochondrial DNA depletion Syndrome
RARS2	Arginyl-tRNA synthetase 2, mitochondrial	Pontocerebellar hypoplasia
SCO2	SCO cytochrome oxidase deficient homolog 2 (yeast)	Leigh syndrome
SDHA	Succinate dehydrogenase complex, subunit A, flavoprotein (Fp)	Leigh syndrome
SURF1	Surfeit 1	Leigh syndrome
TACO1	Translational activator of mitochondrially encoded cytochrome c oxidase I	Leigh syndrome
TMEM70	Transmembrane protein 70	Encephalocardiomyopathy
VDAC1	voltage-dependent anion channel 1	VDAC deficiency

	(d) Mucopolysaccharidosis (MPS) and mucolipidosis (MLP) (15 genes)	

ARSB	Arylsulfatase B	MPS 6 (Maroteaux-Lamy syndrome)
GALNS	Galactosamine (N-acetyl)-6-sulfate sulfatase	MPS 4A (Morquio syndrome)
GLB1	Galactosidase, beta 1	GM1-gangliosidosis
GNPTAB	N-acetylglucosamine-1-phosphate transferase, alpha and beta subunits	Mucolipidosi 2 (I cell disease) and 3A
GNPTG	N-acetylglucosamine-1-phosphate transferase, gamma subunit	Mucolipidosi 3C
GNS	Glucosamine (N-acetyl)-6-sulfatase	MPS 3D (Sanfilippo D syndrome)
GUSB	Glucuronidase, beta	MPS 7 (Sly syndrome)
HGSNAT	Heparan-alpha-glucosaminide N-acetyltransferase	MPS 3C (Sanfilippo C syndrome)
HYAL1	Hyaluronoglucosaminidase 1	MPS 9
IDS	Iduronate 2-sulfatase	MPS 2 (Hunter syndrome)
IDUA	Iduronidase, alpha-L-	MPS 1H (Hurler syndrome)
MCOLN1	Nucolipin 1	Mucolipidosi 4
NAGLU	N-acetylglucosaminidase, alpha	MPS 3B (Sanfilippo B syndrome)
SGSH	N-sulfoglucosamine sulfohydrolase	MPS 3A (Sanfilippo A syndrome)
SUMF1	Sulfatase modifying factor 1	Multiple sulfatase deficiency

	(e) Peroxisome biogenesis disorders (PBD) (9 genes): Zellweger syndrome (ZWS): neonatal adrenoleukodystrophy (NALD): infantile refsum disease (IRD): rhizomelic chondrodysplasia punctata type 1 (RCDP1)	

PEX1	Peroxisomal biogenesis factor 1	ZWS-NADL-IRD
PEX2	Peroxisomal biogenesis factor 2	ZWS-IRD
PEX3	Peroxisomal biogenesis factor 3	ZWS
PEX5	Peroxisomal biogenesis factor 5	ZWS-NADL
PEX6	Peroxisomal biogenesis factor 6	ZWS
PEX7	Peroxisomal biogenesis factor 7	RCDP1
PEX12	Peroxisomal biogenesis factor 12	ZWS
PEX14	Peroxisomal biogenesis factor 14	ZWS
PEX26	Peroxisomal biogenesis factor 26	ZWS-NADL-IRD

**Table 8:** Comparison of commercially available sequencing platforms.

	(a) Massive parallel clonal amplification with optical detection		
	Roche 454	Life Technologies SOLiD	Illumina

Library amplification	emPCR*	emPCR*	On glass
Sequencing	Incorporation of unlabeled dNTPs	Ligase-mediated addition of fluorescent oligoNTPs (2bp)	Incorporation of end-blocked fluorescent dNTPs
Detection	Light emission from release of PPi	Fluorescence emission from ligated dye-labeled oligoNTPs	Fluorescence emission from incorporated labeled oligoNTPs
Progression	Unlabeled dNTPS added in base-specific fashion	Chemical cleavage removes dye and oligoNTP	Chemical cleavage fluorescent dye and blocking group
Errors	Insertion/deletion	End of read	End of read
Length	400 bp	75 bp	150 bp
Overall yield/run	500 Mbp	>100 Gbp	200 Gbp

	(b) Fluorescent and semiconductor single molecule sequencing		

	Helicos	Pacific biosystem	Iontorrent

Library amplification	N/A-tSMS**	N/A-SMRT*** sequencing	Optional PCR
Sequencing	Incorporation of fluorescent labeled dNTPs	Polymerase incorporation terminal phosphate labeled dNTPs	Polymerase incorporation of dNTPs releases H^+^
Detection	Laser-induced emission from incorporated dNTP	Real time detection of fluorescent dye in polymerase active site	Semiconductor ion sensor detects H^+^ released during dNTPs incorporation
Progression	Chemical cleavage of dNTP fluorescent group	N/A fluorescent dyes are removed as PPi with dNTPs incorporation	H^+^ signal during each dNTP incorporation is converted in voltage signal
Errors	Insertion/deletion	Insertion/deletion	Insertion/deletion
Length	35 bp	1000 bp	200–400 bp
Overall yield/run	21–37 Gbp	>100 Gbp	1 Gbp

*emPCR (emulsion PCR) is an amplification method where DNA library fragments are mixed with beads and PCR reagents in an oil emulsion that allows massive amplification of bead-DNA in a single reaction.

**tSMS: true Single Molecule Sequencing.

***SMRT (Single Molecule Real Time)

bp: base pair, Mbp: Mega base pair (10^6^ bp), Gbp: Giga base pair (10^9^ bp), dNTP: deoxynucleotide-tri-phosphate, PPi: pyrophosphate.

**Table 9:** Epilepsy diagnostic panels.

Subpanels With Homogeneous Clinical Entities	Table	Number of genes
Myoclonic epilepsy, febrile seizures, absences	1	37
Encephalopathies	3	30
X-linked mental retardation (XLMR)	4(a)	25
Joubert syndrome	4(b)	10
Lissencephaly and polymicrogyria	4(c)	18
Severe Microcephaly and pontocerebellar hypoplasia	4(d)	22
Walker-Warburg syndrome	4(e)	6
Holoprosencephaly	4(f)	8
Leukodystrophies	5(a)	20
Migraine	5(b)	6
Disorders of the Ras-MAPK pathway	5(c)	13
Hyperekplexia for defective glycine neurotransmission	5(d)	5
Neuronal migration disorders	5(e)	31
Inherited errors of metabolism	6	49
Congenital disorder of glycosylation (CDG)	7(a)	23
Neuronal ceroid lipofuscinosis (NCL)	7(b)	8
Defects of mitochondrial metabolism including coenzyme Q deficiency	7(c)	35
Mucopolisaccaridosis and mucolipidosis	7(d)	15
Peroxisome biogenesis disorders (PBD)	7(e)	9
Syndromic epilepsy	2	47
